# Donor Age of Human Platelet Lysate Affects Proliferation and Differentiation of Mesenchymal Stem Cells

**DOI:** 10.1371/journal.pone.0037839

**Published:** 2012-05-25

**Authors:** Michael Lohmann, Gudrun Walenda, Hatim Hemeda, Sylvia Joussen, Wolf Drescher, Stefan Jockenhoevel, Gabriele Hutschenreuter, Martin Zenke, Wolfgang Wagner

**Affiliations:** 1 Department for Stem Cell Biology and Cellular Engineering, Helmholtz-Institute for Biomedical Engineering, RWTH Aachen University Medical School, Aachen, Germany; 2 Department for Orthopedics, RWTH Aachen University Medical School, Aachen, Germany; 3 Department of Applied Medical Engineering, Helmholtz-Institute for Biomedical Engineering, RWTH Aachen University, Aachen, Germany; 4 Institute for Transfusion Medicine, RWTH Aachen University Medical School, Aachen, Germany; 5 Institute for Biomedical Engineering – Cell Biology, RWTH Aachen University Medical School, Aachen, Germany; Clinica Universidad de Navarra, Spain

## Abstract

The regenerative potential declines upon aging. This might be due to cell-intrinsic changes in stem and progenitor cells or to influences by the microenvironment. Mesenchymal stem cells (MSC) raise high hopes in regenerative medicine. They are usually culture expanded in media with fetal calf serum (FCS) or other serum supplements such as human platelet lysate (HPL). In this study, we have analyzed the impact of HPL-donor age on culture expansion. 31 single donor derived HPLs (25 to 57 years old) were simultaneously compared for culture of MSC. Proliferation of MSC did not reveal a clear association with platelet counts of HPL donors or growth factors concentrations (PDGF-AB, TGF-β1, bFGF, or IGF-1), but it was significantly higher with HPLs from younger donors (<35 years) as compared to older donors (>45 years). Furthermore, HPLs from older donors increased activity of senescence-associated beta-galactosidase (SA-βgal). HPL-donor age did not affect the fibroblastoid colony-forming unit (CFU-f) frequency, immunophenotype or induction of adipogenic differentiation, whereas osteogenic differentiation was significantly lower with HPLs from older donors. Concentrations of various growth factors (PDGF-AB, TGF-β1, bFGF, IGF-1) or hormones (estradiol, parathormone, leptin, 1,25 vitamin D3) were not associated with HPL-donor age or MSC growth. Taken together, our data support the notion that aging is associated with systemic feedback mechanisms acting on stem and progenitor cells, and this is also relevant for serum supplements in cell culture: HPLs derived from younger donors facilitate enhanced expansion and more pronounced osteogenic differentiation.

## Introduction

Regeneration of human tissues is accomplished by adult stem and progenitor cells. The regenerative potential decays upon aging and therefore, aging can be interpreted as loss of function in somatic stem cells [1]. Mesenchymal stem cells (MSC) resemble precursor cells for various mesodermal lineages such as adipocytes, chondrocytes and osteocytes [2], [3]. They can be isolated from many tissues and culture expanded over several passages. However, MSC preparations are heterogeneous and only a subset possesses multilineage-differentiation potential - therefore it may be more appropriate to alternatively use the term “mesenchymal stromal cells” [4]–[6].

The ease of culture expansion of MSC has raised high hopes for a broad range of applications in regenerative medicine [7]. On the other hand, culture conditions have major impact on the composition of cell preparations and their functional potency [8]. The potency is not necessarily reflected by proliferation or *in vitro* differentiation potential – it is rather important how these cell preparations perform under *in vivo* conditions of a specific clinical setting. Fully defined synthetic culture media would be preferable for standardization of therapeutic products. However, despite intensive research serum additives usually need to be added. To date, fetal calf serum (FCS; alternatively termed “fetal bovine serum” [FBS]) is the most commonly used serum supplement. In the light of clinical application there is emerging interest to avoid the use of FCS due to the risk of xenogenic immune reactions, infections with bovine viruses or prions and a high batch-to-batch variation [9], [10]. In contrast, various types of human supplements have been shown to serve as substitute for animal serum in clinical-scale expansion of MSC such as 1) human serum [11], 2) human thrombin-activated platelet releasate in plasma [12], 3) platelet rich plasma (PRP) [13] or 4) human platelet lysate (HPL). The later can be generated from platelet units which derived from apheresis. HPL has proven as very efficient substitute for FCS and many laboratories have changed their culture conditions accordingly [12], [14]–[16].

Human platelet lysate is generated by a simple freeze-thaw procedure of platelet units – thus, it is rich in growth factors released from the platelet fraction. These growth factors include platelet derived growth factor (PDGF), transforming growth factor beta 1 (TGF-β1), insulin like growth factor (IGF-1) and basic fibroblast growth factor (bFGF) [17]–[19]. This might explain the consistently higher proliferation and culture expansion rate of MSC in HPL media in comparison to FCS [12], [13], [16], [20], [21]. To minimize batch-to-batch variation, individual HPLs are usually pooled but this entails higher risks of immunological consequences or viral infections as each unit is a potential source of pathogens [22]. Recently, we have demonstrated that there is some variation in 10 HPLs derived from single donor apheresis samples [21]. HPLs from platelet units with higher platelet counts or with higher PDGF concentrations seemed to enhance culture expansion of MSC. Reanalysis of the data indicated that there might also be an age-associated effect on proliferation. However, due to the low number of samples, no statistical analysis was feasible.

Indeed, age-associated effects of serum supplements were already proposed in 1921 when cell culture methods were still in its infancy [23], [24] and more recently this was supported in breast cancer cell lines [25] and in rat MSC [26]. On the other hand, enhanced cell growth with human serum from younger donors was not observed in several other studies [27]–[29]. Therefore, the question remains if donor age of HPL affects MSC expansion or *in vitro* differentiation.

In the present study we sought to further address variation between individual HPLs and to identify relevant parameters for culture expansion of MSC from either bone marrow or adipose tissue. We have directly compared 31 individual HPLs with regard to proliferation, fibroblastoid colony-forming unit (CFU-f) frequency, expression of SA-βgal, immunophenotype as well as adipogenic and osteogenic differentiation. Subsequently, the variation in MSC growth was correlated with concentrations of various growth factors and hormones - however, the most significant association was observed with HPL-donor age.

## Materials and Methods

### Ethics Statement

This study was approved by the Ethic Committee of the University of Aachen and all samples were isolated after written consent. MSC derived from bone marrow of the *caput femoris* taken during hip replacement surgery (permit number EK128/09). MSC from adipose tissue were isolated from lipoaspirates of healthy young patients undergoing cosmetic surgery (permit number EK163/07).

### Generation of Human Platelet Lysate

Human platelet lysates (HPLs) were generated from platelet units that were harvested from individual healthy donors by apheresis using the Trima Accel Collection System (CaridianBCT, Garching, Germany). The donors are examined closely - they have to be in healthy condition and between 18 and 65 years old (therefore the available age-range for HPL is limited). Thrombocyte concentrates have a platelet content of 2.0 to 4.2×10^11^ platelets in 200 mL plasma supplemented with Acid-Citrate-Dextrose (ACD) (1∶11 v/v). After their expiry date (five days after harvesting) platelet units were frozen twice at −80°C and re-thawed at 37°C. To remove membrane fragments, lysates were centrifuged at 2600×*g* for 30 min and the supernatant was filtered through 0.2 µm GD/X PVDF filters (Whatman, Dassel, Germany). HPLs were stored at −80°C until use and 2 U/mL heparin was added to avoid gelatinization. In this study we have used 31 HPLs of 25 to 57 year old donors. Distribution among blood types was A: 17, B: 3, AB: 2, and 0∶9 samples. In order to exclude any possible effect of gender only male samples were considered.

### Quantification of Growth Factors and Hormones in HPL

The amount of PDGF-AB in the HPL was assayed with the Quantikine Human PDGF-AB enzyme-linked immunoabsorbent assay (ELISA; R&D Systems, Abingdon, UK). bFGF was analyzed with the Quantikine Human bFGF ELISA kit (R&D Systems). TGF-β1 was assayed with the RayBio Human TGF-β1 ELISA kit (Raybiotech, Norcross, GA, USA) after activation of latent TGF-β1 to the immunoreactive form according to manufacturer’s instructions. Human leptin was assessed with the Human Leptin ELISA (Biovendor, Brno, Czech Republic). ELISAs were quantified by measuring the absorbance with a plate reader (Tecan Infinite M200; Tecan Deutschland GmbH, Crailsheim, Germany) at 450 nm with a reference wavelength of 540 nm. 1,25 vitamin D3 was analyzed with the IDS 1,25 Dihydroxy Vitamin D enzyme immunoassay (EIA, IDA Ltd, Boldon, UK). IGF-1 and growth hormone were assayed with solid-phase, enzyme-labeled chemiluminescent assays (Immulite 2000 system, Siemens Healthcare Diagnostic Products Ltd, Llanberis, UK). These assays were quantified by measuring the absorbance with the Wallac Victor^2^ plate reader (PerkinElmer, Waltham, MA, USA). Estradiol and parathormone were analyzed with electrochemiluminescence immuno assays (ECLIA) with a cobas e 411 system (Roche Diagnostics GmbH, Mannheim, Germany).

### Cell Culture Conditions

Bone marrow (BM)-derived MSC were isolated from the *caput femoris* from patients undergoing hip replacement surgery as described before [21], [30] (n = 11; Table 1). It needs to be taken into account that these are predominantly elderly patients who are more susceptible to osteoporosis and therefore the cellular composition of these may differ from bone marrow aspirates of healthy younger donors [31], [32]. Briefly, bone fragments were flushed with PBS and mononuclear cells (MNC) were subsequently isolated by density gradient on Biocoll (Biochrom AG, Berlin, Germany).

**Table 1:** *MSC donor age and gender.*

MSC donor	age	gender
Bone marrow derived MSC:		
BM-MSC 1	70	female
BM-MSC 2	73	female
BM-MSC 3	55	female
BM-MSC 4	65	male
BM-MSC 5	65	female
BM-MSC 6	76	male
BM-MSC 7	63	female
BM-MSC 8	68	male
BM-MSC 9	69	female
BM-MSC 10	48	female
BM-MSC 11	77	female
Adipose tissue derived MSC:		
AT-MSC 1	19	male
AT-MSC 2	35	male
AT-MSC 3	53	male

Adipose tissue (AT)-derived MSC were isolated from lipoaspirates that were washed in 9 g/L NaCl, centrifuged at 300×*g* for 10 minutes and the middle layer was subsequently digested with 2 g/L collagenase type I (Biochrom; specific activity 125–250 Mandl Units/mL of powder substance) for 45 min at 37°C on a horizontal rotary shaker [20]. The digested tissue was passed through a 100 µm cell strainer (BD Biosciences, Franklin Lake, NJ, USA) and re-suspended in corresponding culture media. In this study we used AT-MSC from three different donors (Table 1).

Cell culture was performed in polystyrene cell culture dishes (Greiner, Kremsmünster, Austria). Culture medium consisted of Dulbeccós Modified Eagles Medium-Low Glucose (DMEM-LG; PAA, Pasching, Austria) with 2 mM L-glutamine (Sigma-Aldrich, St. Louis, MO, USA) and 100 U/mL penicilline/streptomycine (pen/strep; Lonza, Basel, Switzerland). Culture medium was supplemented with 10% FCS (HyClone, Bonn, Germany) or 10% HPL of individual donors as indicated in the text. The first medium exchange was performed after 48 hours to remove erythrocytes and other non-adherent cells. Thereafter, medium was changed twice per week.

Some experiments were performed with MSC which were freshly isolated from bone marrow using different HPLs (CFU-f analysis and immunophenotype). For other experiments the MSC were expanded with the same HPL supplement for several passages to provide a comparable starting population for subsequent comparison of different HPLs (proliferation assays, long-term expansion curves and SA-βgal activity). When reaching 80% confluence, cells were trypsinized, counted in a Neubauer counting chamber (Brand, Wertheim, Germany) and replated at 1,000 cells/cm^2^. Cell Population doublings per passage (PDP) and cumulative population doublings (CPD) were calculated from the time when culture media with the different HPL supplements were tested in parallel (passage 2 for long-term growth curves) and calculated as described in our previous work [20], [33].

### Proliferation Assay

Cell proliferation was assessed via the Thiazolyl Blue Tetrazolium Bromide (MTT) assay [34]. Cells of passage 4 to passage 7 were seeded on a 96-well plate (100 cells per well) with different serum supplements as described above. After 7 days, cells were washed and incubated with 1 mM 3-(4,5-Dimethyl-2-thiazolyl)-2,5-diphenyl-2H-tetrazolium bromide (MTT, Sigma-Aldrich) in PBS for 3.5 hours. The excess solution was discarded and crystals were resolved in 4 mM HCl in isopropanol (both from Roth, Karlsruhe, Germany). Optical density was measured at a wavelength of 590 nm with a reference wavelength of 620 nm using the Tecan Infinite M200 plate reader.

### Analysis of Senescence-associated β-galactosidase Activity

Senescence-associated β-galactosidase (SA-βgal) activity was assessed by a fluorescence-based assay using flow cytometry [35]. MSC of either passage 3, 5, or 6 (1,000 cells per cm^2^) were cultured in parallel with different HPLs. After 5 to 6 days sub-confluent cells were incubated with 100 nM Bafilomycin A1 (Sigma-Aldrich) for 1 hour to alkalize the lysosomes. Cells were then incubated for 2 hours with 33 µL of 2 mM 5-dodecanoylaminofluorescein-di-β-D-galactopyranoside (C12FDG, Invitrogen, Carlsbad, CA, USA), washed thoroughly and analyzed with a FACSCanto II (BD Biosciences). Data were analyzed with WinMDI (Windows Multiple Document Interface for Flow Cytometry) software (TreeStar, Ashland, OR, USA).

### Analysis of Colony Formation

Freshly isolated MNC were counted in a Neubauer counting chamber and seeded in different dilutions on 24-well plates (250,000; 100,000; 50,000; 20,000; 6,000; 2,000 and 1,000 cells per well). After 8 days, the plastic adherent colonies were stained with crystal violet (Fluka Analytical, Buchs, Switzerland). We have chosen those dilutions for further analysis which facilitated clear separation of fibroblastic colony-forming units (CFU-f). Colonies consisting of more than 20 cells were taken into account. Subsequently, colony size was reanalyzed in cryopreserved 24-well plates by counting of cells per colony.

### Immunophenotypic Analysis

Immunophenotypic analysis was performed during the primary passage upon culture-isolation of MSC in different HPLs. Primary culture was performed for three weeks to generate enough cells and non-adherent hematopoietic cells were washed away by rigid medium exchanges. Expression of surface markers was analyzed with a FACSCanto II (BD Biosciences) using the following monoclonal antibodies: mouse anti-human CD14-APC (clone M5E2), CD29-PE (clone MAR4), CD31-PE (clone WM59), CD34-APC (clone 8G12), CD45-APC (clone HI30), CD73-PE (clone AD2), CD90-APC (clone 5E10), CD140α-PE (clone αR1) (all from BD Biosciences), CD105-FITC (clone MAR-226; ImmunoTools, Friesoythe, Germany), CD271-APC (clone ME20.4-1.H4; Miltenyi Biotech, Bergisch Gladbach, Germany). Data were analyzed with WinMDI software.

## *In vitro* Differentiation

Osteogenic and adipogenic differentiation of BM-MSC was performed as described before [2], [30]. In brief, culture media was changed to adipogenic or osteogenic differentiation media supplemented with either 10% FCS or 10% of the corresponding HPL to assess the impact of individual HPLs on differentiation.

Adipogenic differentiation media consisted of DMEM-High Glucose with 2 mM L-glutamine, 1 µM dexamethasone, 0.5 mM 1-methyl-3-isobutylxanthin (IBMX), 10 µg/mL insulin (all from Sigma-Aldrich) and 100 U/mL pen/strep (Lonza). Medium was changed twice per week. After three weeks, fat droplet formation upon adipogenic differentiation was stained with Oil Red O (Sigma-Aldrich), extracted with 99% isopropanol (Roth) and quantified with the Tecan Infinite M200 plate reader at a wavelength of 550 nm with a reference wavelength of 650 nm. Alternatively, wells were stained with the green fluorescent dye BODIPY (4,4-difluoro-1,2,5,7,8-pentamethyl-4-bora-3a,4a-diaza-s-indacene) and counterstained with DAPI (4′,6-Diamidin-2-phenylindol; both Molecular Probes, Eugene, Oregon, USA) as described previously [36]. Microscopic images were taken with a Leica DM IL HC fluorescence microscope (Leica, Wetzlar, Germany).

Osteogenic differentiation media consisted of DMEM-LG (PAA) with 2 mM L-glutamine, 100 nM dexamethasone, 200 µM L-ascorbic acid-2-PO_4_, 10 mM β-glycerophosphate (all Sigma-Aldrich) and 100 U/mL pen/strep (Lonza). Medium was changed twice per week. After two weeks, osteogenic differentiation was analyzed upon staining with Alizarin S Red with the Tecan Infinite M200 plate reader at a wavelength of 550 nm with a reference wavelength of 650 nm. Additionally, images were taken with a Leica DM IL HC microscope.

### Statistics

Results are expressed as mean ± standard deviation or as a box-and-whisker plot where the whiskers represent the minima and maxima. Linear regression analysis was performed with Excel (Microsoft cooperation). To estimate the probability of differences we have adopted the Student’s T-test. Probability value of P<0.05 denoted statistical significance.

## Results

### The Proliferation Rate of MSC Varies between Platelet Lysates

Thirty-one single donor derived HPLs were compared for culture expansion of MSC from bone marrow (BM) or adipose tissue (AT). MSC at passage 4 to 7 were seeded in parallel with culture media which were supplemented with 10% of one of these HPLs. As a reference, we have used 10% FCS. After 7 days proliferation was measured with the MTT assay. AT-MSC proliferation was about 5.1-fold higher with HPL than with FCS (P = 0.01), whereas this growth stimulation was less pronounced in BM-MSC (1.4-fold; P = 0.27). The proliferation rate varied in culture media supplemented with different HPLs, and these differences were consistently observed with different MSC preparations (BM-MSC, n = 4; AT-MSC, n = 3).

Next, we aimed to identify relevant factors for MSC growth. The proliferative effect of HPL correlated only moderately with platelet counts of the HPL donors (Figure S1A,B). Concentrations of PDGF-AB, bFGF, TGF-β1 and IGF-1 were measured in the HPLs using commercially available ELISA kits. These growth factors revealed some variation between HPLs - however, none of them correlated significantly with the proliferation of MSC (Figure S1C–J). Furthermore, there was no association between blood groups of HPL and cell growth. Therefore, these parameters do not seem to be of major relevance for MSC growth.

### Platelet Lysate of Young Donors Increases Proliferation

**Figure 1:**
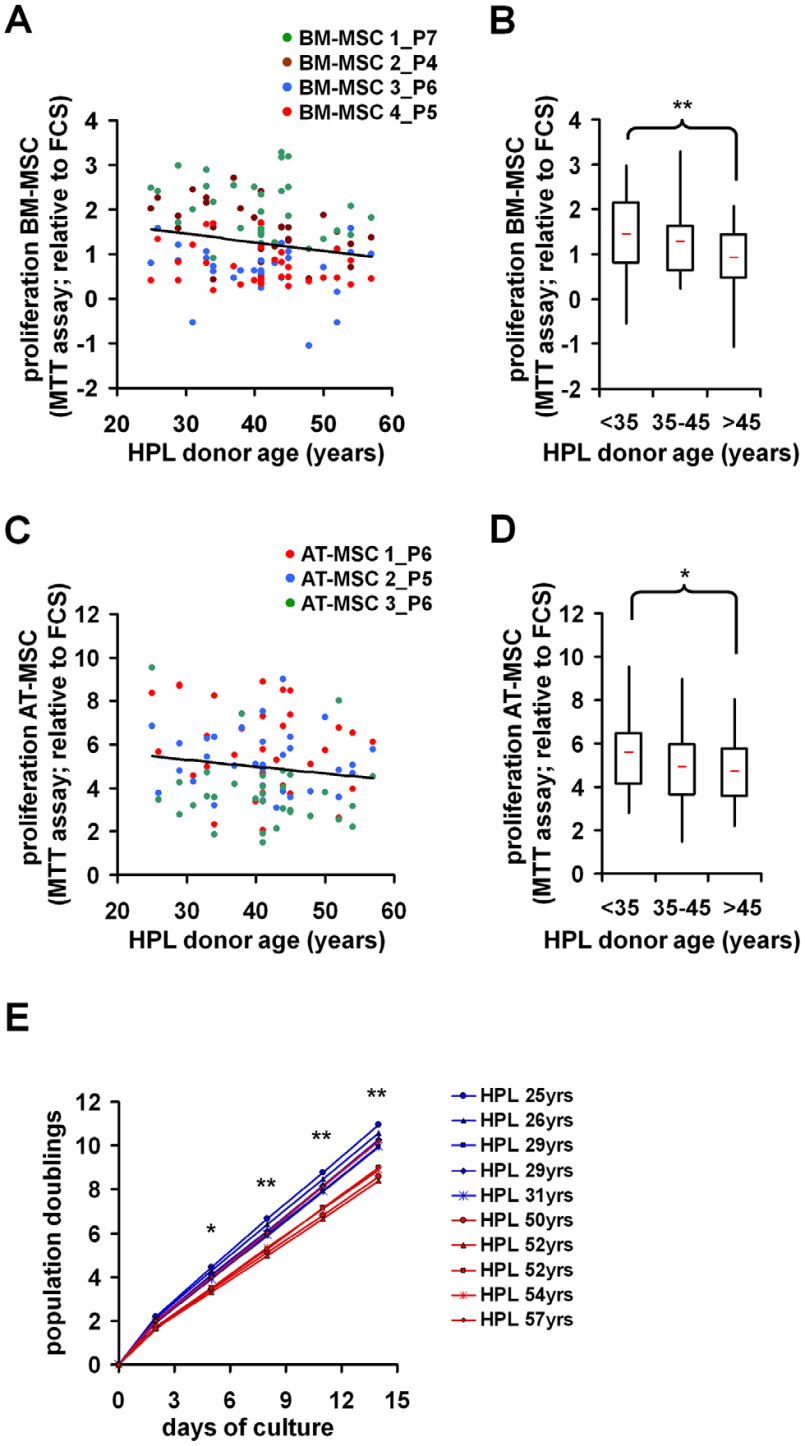
Proliferation is associated with HPL-donor age. Mesenchymal stem cells from bone marrow **(**BM-MSC; n = 4) or adipose tissue (AT-MSC; n = 3; passage numbers are indicated) were cultured for seven days with media supplemented with different HPLs. Subsequently, proliferation was assessed via MTT assay and normalized to cell growth with FCS. Proliferation was higher in media supplemented with HPLs of younger donors in all biological replica derived from bone marrow (A,B) and adipose tissue (C,D). For statistical analysis HPLs were classified in three age-groups: <35 years, 35 to 45 years, and >45 years (B,D). MSC at passage 2 were simultaneously cultured for 5 additional passages with HPL derived from 5 young and 5 older donors (E). **P<0.01; *P<0.05.

Subsequently, we analyzed if the variation in MSC growth correlated with the donor age of individual HPLs. Overall, the growth promoting effect of HPLs decreased upon aging, and this was observed in different MSC preparations from bone marrow (n = 4) and adipose tissue (n = 3; Figure 1). These differences were significant comparing the lower and the upper quartile Q1 and Q3 (Q1: <35 years, n = 8, mean age 30; Q2∶35 to 45 years, n = 16, mean age 42; and Q3: >45 years, n = 7, mean age 52; BM-MSC: P = 0.008; AT-MSC: P = 0.05). To exclude the effect that MSC donor age may have on cell growth rate, each cell preparation was simultaneously cultured with the 31 different HPL supplements. In average, proliferation of MSC with HPLs of younger donors was about 1.4-fold higher than with HPLs of elderly donors.

Subsequently, we have analyzed if higher proliferation with HPL of younger donors is maintained over several passages. To this end, MSC at passage 2 were further expanded in parallel with either five HPLs of young donors (25–31 years) or five HPLs of older donors (50–57 years). After five additional passages MSC gained in average 1.3 additional cumulative population doublings when they were cultured with HPL of younger donors as compared to older donors (10.33 CPD *versus* 8.99 CPD; P<0.01). The deviance increased from passage to passage (Figure 1E). These results demonstrate that age-associated changes in HPL supplements are relevant for proliferation of MSC.

### Senescence-associated β-galactosidase Activity Increases with HPL of Older Donors

**Figure 2:**
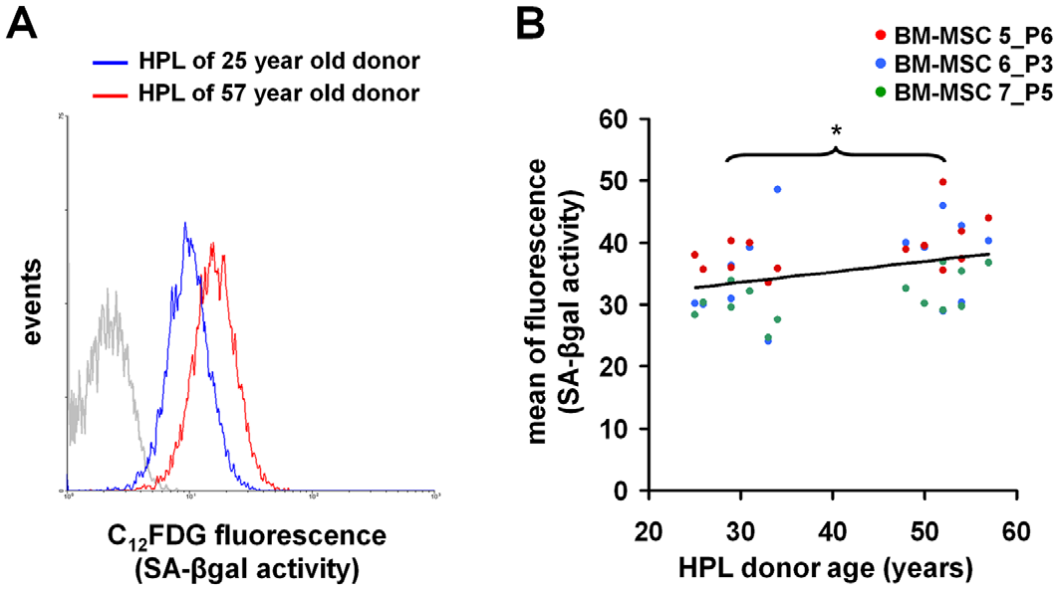
HPL-donor age is associated with activity of senescence-associated β-galactosidase. MSC were cultivated for 5–6 days in HPL media of younger (<35 years; n = 8) or older donors (>45 years, n = 7). C_12_FDG, a fluorogenic substrate of SA-βgal was subsequently analyzed by flow cytometry. A representative histogram of fluorescence intensity is demonstrated (grey line: autofluorescence; blue line: youngest HPL donor; red line: oldest HPL donor). Comparison of mean fluorescence intensity between the age-groups revealed higher SA-βgal activity with HPL of older donors (B). *P<0.05.

HPL-donor age might also be relevant for cellular aging of MSC in culture. Therefore, we cultured BM-MSC with HPL of young (<35 years; n = 8) or older donors (>45 years; n = 7) for five to six days and determined activity of senescence-associated beta-galactosidase (SA-βgal). This lysosomal enzyme permits identification of senescent cells in culture and can be quantified by flow cytometry [35]. Overall, the mean signal intensity of the fluorogenic substrate 5-dodecanoylaminofluorescein di-β-D-galactopyranoside (C_12_FDG) significantly increased with HPL of elderly donors (P = 0.02; Figure 2). Thus, HPL donor age is associated with activity of this biomarker for cellular senescence.

### HPL-donor Age does not Affect CFU-f

**Figure 3:**
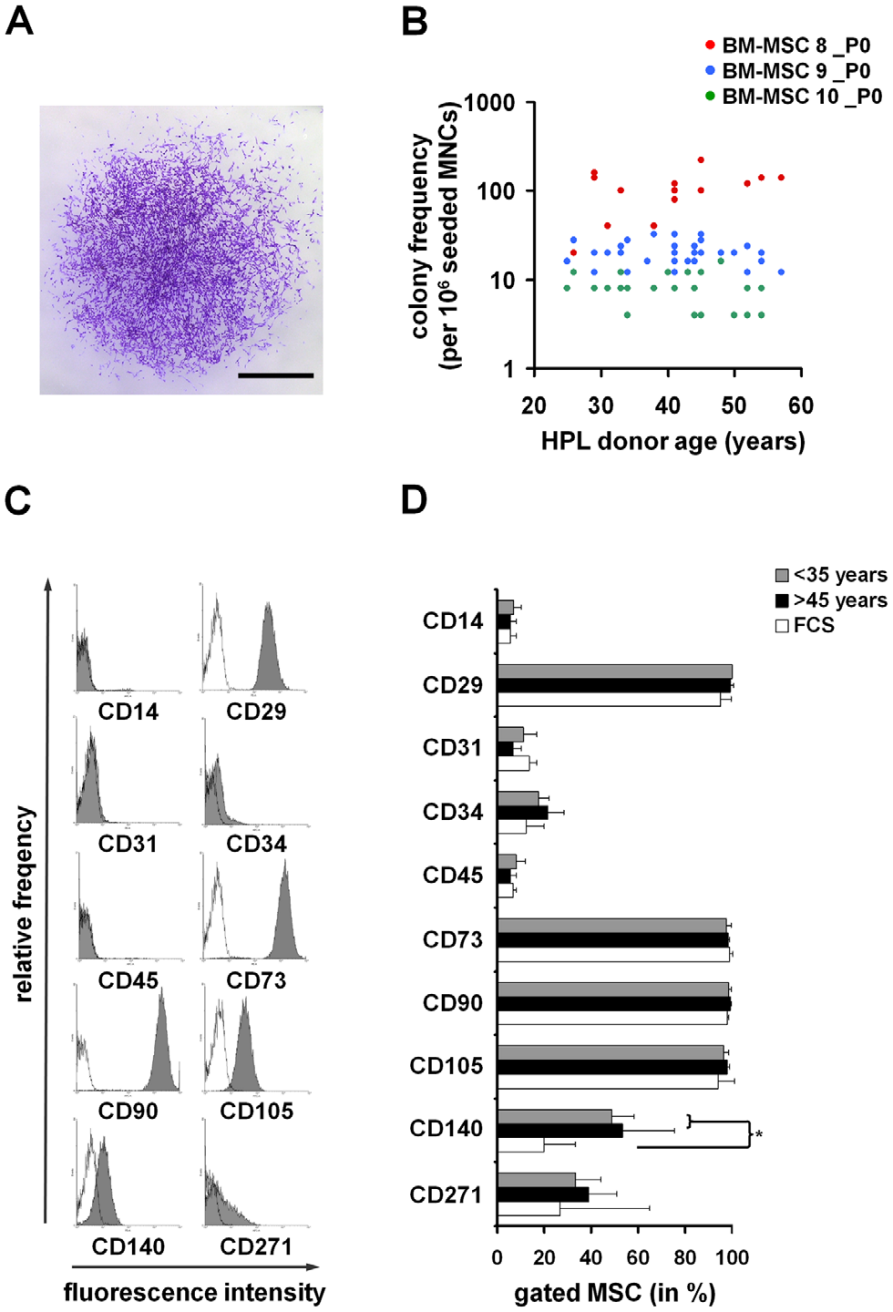
Fibroblastoid colony forming unit (CFU-f) frequency and immunophenotype. Mononuclear cells were isolated from bone marrow and seeded in dilution series with media containing 31 different HPLs or FCS – these experiments were performed with three individual MSC preparations. CFU-f frequency was assessed after 7 days via crystal violet staining (A). CFU-f frequency varied between different MSC preparations, but there was no association with HPL-donor age within either of the MSC preparations (B). Subsequently, cell preparations were expanded for one additional passage. Flowcytometry revealed the commonly described surface marker profile for MSC (C; autofluorescence =  black line). HPLs derived from younger donors (<35 years; n = 5), or HPLs derived from older donors (>45 years; n = 5) did not have significant impact on the immunophenotype (D). *P<0.05; scale bar = 500 µm.

Subsequently, we analyzed whether the initial frequency of fibroblastoid colony units (CFU-f) is affected by different HPLs. BM-MSC were isolated and cultured in parallel in culture media containing the 31 different HPLs. CFU-f frequency was determined after 8 days. Overall, CFU-f frequency was about 3.3-fold higher with HPL as compared to FCS (P = 0.05). There was notoriously some variation between different bone marrow samples (n = 3) and between different HPLs. No correlation was observed between CFU-f frequency and HPL-donor age (Figure 3A,B). Overall, colonies were larger if cultured with HPL of younger donors (Q1: mean colony size 186 cells) as compared to HPL of older donors (Q3: mean colony size 139 cells) although these results were not significant (P = 0.09). Furthermore, CFU-f frequency did not correlate to concentrations of PDGF-AB, bFGF, TGF-β1 and IGF-1 in the HPLs (data not shown).

### Immunophenotypic Analysis of MSC

MSC are commonly characterized by a panel of surface markers [6]. To address whether HPL-donor age has impact on the immunophenotype of MSC, cells were isolated in parallel from fresh bone marrow with FCS, HPLs of young (<35 years; n = 5) or older donors (>45 years; n = 5). Immunophenotypic analysis revealed hardly any contamination of remaining CD45^+^ blood cells. All MSC preparations revealed the typical surface marker profile for MSC: CD29^+^, CD73^+^, CD90^+^, CD105^+^, CD14^−^, CD31^−^, CD34^−^, CD45^−^, CD140^+^, and CD271^low^ (Figure 3C). The percentage of positive cells was not affected by HPL of young or elderly donors. Notably, expression of CD140α (PDGF-Receptor α) was higher in MSC cultured with HPL as compared to those cultured with FCS (P = 0.03; Figure 3D).

### *In vitro* Differentiation of MSC

**Figure 4:**
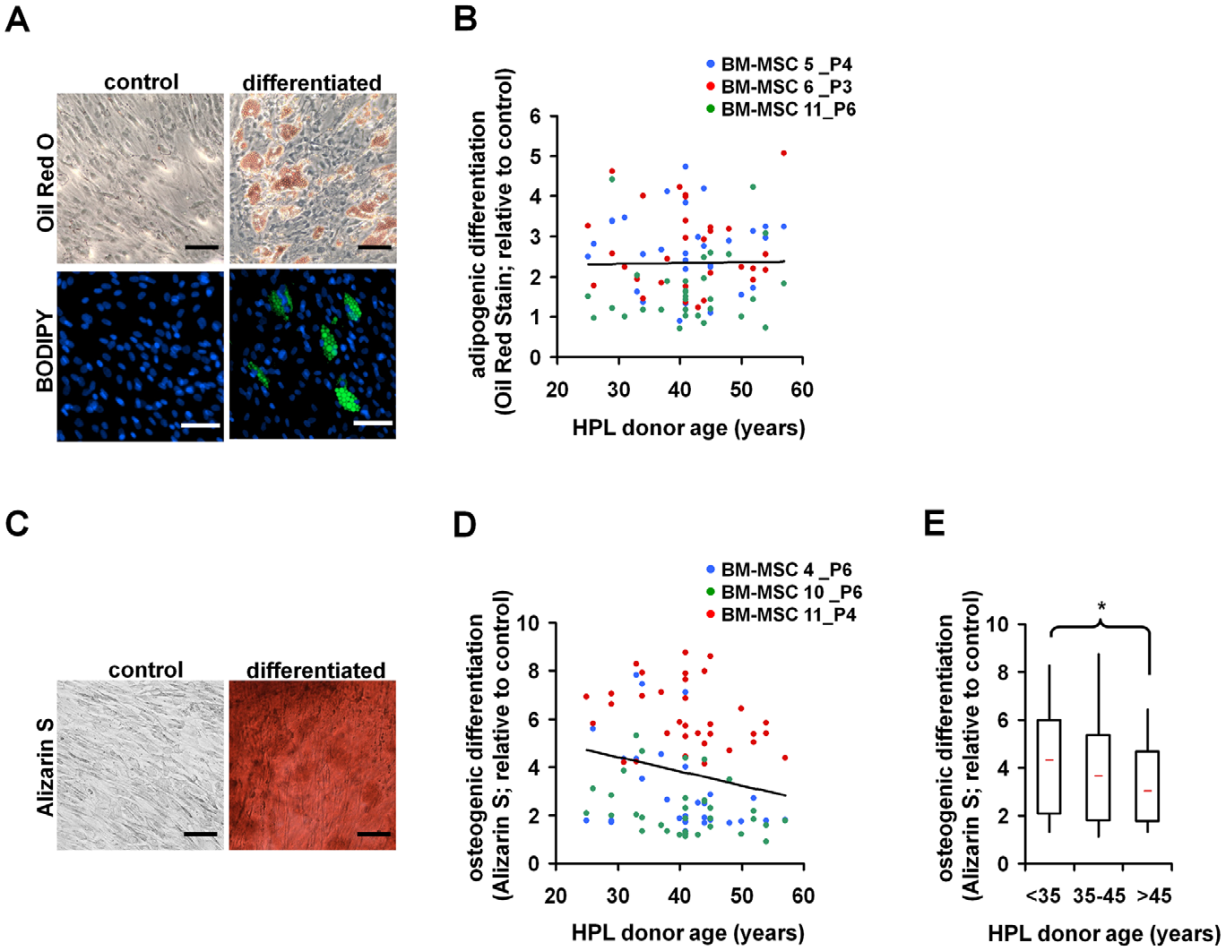
In vitro differentiation of MSC in relation to HPL-donor age. Bone marrow derived MSC were differentiated towards adipogenic (A,B) or osteogenic lineage (C–E) using induction media supplemented with different HPLs. After 3 weeks adipogenic differentiation was assessed by staining of lipid droplets with Oil Red O or BODIPY (nuclei were counterstained with DAPI) in comparison to non-differentiated controls (A). Quantitative analysis did not reveal any association between adipogenic differentiation and HPL-donor age (B; n = 3). Osteogenic differentiation was analyzed after two weeks by staining of calcium phosphate precipitates with Alizarin S (C). HPLs of younger donors stimulated osteogenic differentiation more than HPLs of elderly donors (D; n = 3). For statistical analysis HPLs were classified in three age groups: <35 years, 35 to 45 years, and >45 years (E). *P<0.05; scale bar = 100 µm.

Subsequently, we assessed if the *in vitro* differentiation potential of BM-MSC was influenced by the different HPLs. Adipogenic differentiation was analyzed by staining of lipid droplets with Oil Red O and quantification on a plate reader. Alternatively, the number of differentiated cells was determined with BODIPY staining. There were reproducible differences in adipogenic differentiation with different HPLs, but there was no correlation with growth factor concentrations or HPL-donor age (Figure 4A,B).

**Figure 5:**
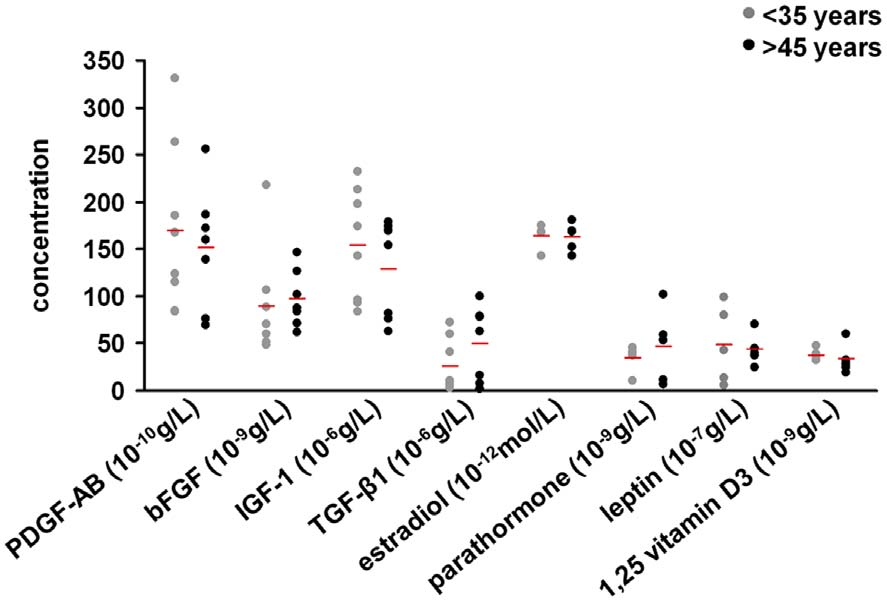
Age-related concentration of growth factors and hormones in HPL media. Concentrations of PDGF-AB, bFGF, IGF-1, TGF-β1, estradiol, parathormone, leptin and 1,25 vitamin D3 were measured in media supplemented with HPL from either younger donors (<35 years; n = 5 to 7) or older donors (>45 years; n = 5 to 8). None of these compounds revealed significant age-associated changes.

Osteogenic differentiation was determined by staining of calcium phosphate precipitates with Alizarin S Red and quantification using a plate reader. Overall, osteogenic differentiation was higher in HPLs derived from younger donors. This notion was supported by classification of HPLs in a younger (Q1, <35 years) and older group (Q3, >45 years; P = 0.04; Figure 5C–E). These results are in line with a previous report indicating that osteogenic differentiation but not adipogenic differentiation is impaired with serum of elderly donors [28].

### Growth Factors and Hormones in HPL

We reasoned that the HPL-donor age-associated differences in growth stimulation, SA-βgal activity and osteogenic induction might correlate with concentrations of specific growth factors or hormones which have been implicated in these processes. Therefore, we have compared PDGF-AB, bFGF, TGF-β1, IGF-1, estradiol, leptin, parathormone and 1,25 vitamin D3 in HPL of young (<35 years) and elderly donors (>45 years). There were no significant age-associated differences in concentrations of these factors (Figure 5).

## Discussion

Cellular communication with an aging environment affects the regenerative potential. This has been suggested by parabiosis pairings of mice with a shared circulatory system. Heterochronic parabioses of young and aged mice continuously provide serum of young animals to their aged counterpart and this rejuvenates activation of aged satellite cells and proliferation of aged liver progenitor cells [37]. Therefore, age-associated changes are not only cell-intrinsically determined, but they are also governed by systemically released factors. Our results indicate that similar mechanisms can also be attributed in cell culture: HPLs from younger donors stimulate proliferation of MSC and enhance their osteogenic differentiation as compared to HPLs from older donors. Notably, also the activity of senescence-associated β-galactosidase increased using HPLs of older donors. This is in line with a recent study of Zhang and co-workers who demonstrated increased proliferation and more SA-βgal positive cells with pooled serum of older rats (64–68 weeks old) as compared to younger rats (3–4 weeks old) [26]. Replicative senescence *in vitro* is certainly not identical with the complex changes during aging of the organism, but there is evidence that molecular changes in long-term culture and aging are related [38], [39]. Zhou and co-workers demonstrated that at passage 2 more MSC are positive for SA-βgal in MSC derived from older than from younger subjects [40]. Thus, the increased SA-βgal activity with HPL of older donors indicates that aging of MSC is enhanced by serum supplements of elderly donors.

In this study, we have always supplemented 10% of either FCS or HPL. We have chosen this concentration as it is often used for standardized expansion procedures [41]. However, FCS and HPL are much different materials and this renders a direct comparison of the two serum supplements at a given concentration difficult. HPL enhanced proliferation 1.4-fold in MSC derived from bone marrow and even 5.1-fold in MSC from adipose tissue as compared to FCS. These results correspond to our previous observations that especially proliferation of AT-MSC is increased with HPL [20], [21] providing evidence that MSC from different tissues are distinct in their nutritional requirements. It is even anticipated that BM-MSC, which are either derived from *caput femoris* or from bone marrow aspirates from the iliac crest, reveal differences in cell growth and differentiation. The growth promoting effect of HPL is probably due to growth factors or other specific compounds which are released from the platelet fraction. It has been controversially discussed whether PDGF-AB is relevant for MSC proliferation [42]–[46]. In our previous work, we have analyzed 10 HPLs with RayBiotech Chemokine membranes and the evaluation revealed that PDGF-AB had the best association with MSC proliferation. However, this is not confirmed by our present study with more precise ELISA measurements and a higher number of HPL samples. PDGF-AB is the most abundant isoform and binds to the receptor-subtypes PDGF-Rαα and PDGF-Rαβ – it does not bind to PDGF-Rββ which has recently been associated with proliferation and preservation of stem cell function in MSC [45], [47]. bFGF, TGF-β1 and IGF-1 have also been suggested to be relevant for MSC growth [17], [18], [48], whereas a growth-stimulatory effect was not observed in other studies [42], [49]. Our study did not reveal significant correlation for any of these growth factors with MSC growth indicating that their concentration is not growth-limiting.

Interestingly, the most abundant correlation for MSC proliferation was observed with HPL-donor age: cell growth was enhanced with serum from younger donors. This was observed with MSC derived from bone marrow of the caput femoris and from adipose tissue. Furthermore, the same tendency was also observed when we reanalyzed our previous data with exclusion of two female samples [21]. On the other hand, age-associated effects of serum supplements were not observed in other studies [27]–[29] which might be due to the use of serum instead of HPL. These effects might also be abrogated with other cell types such as fibroblasts [29], or by examining female serum exclusively [28]. Furthermore, cell preparations change continuously in the course of culture expansion, and therefore HPL-donor age may also exert different effects on very early or very late passages [30], [50]. Notably, HPL-donor age did not influence CFU-f frequency. For this analysis we considered large and very small colonies as well – therefore CFU-f frequency is particularly influenced by the percentage of cells which are capable of surface adherence and outgrowth. In contrast, proliferation is rather reflected by colony size. As expected, colonies consisted of more cells if cultured with HPL of younger donors although this was not significant. The age-associated effect of individual serum supplements would be levelled by pooling of large numbers of HPLs to generate a consistent lot [51]. On the other hand, this might increase the risk of infection with pathogens such as human immunodeficiency virus (HIV) or hepatitis B and C. In contrast to thrombocyte concentrates, which need to be used freshly, it is possible to quarantine frozen-stored HPL [52]. HPL-donors may then be tested after an interval of several months to reduce the risk, but even so it may be safer to use only one or few HPLs.

Several studies have shown an inverse relationship between MSC-donor age and the replicative life span of MSC and fibroblasts [53]–[55]. This effect is usually relatively small with a high variation between different donor samples [32], [56]. In this study, we did not use enough samples to address such inter-individual differences. Either way, the effect of MSC-donor age was not the focus of this study but the effect of HPL-donor age. Therefore, all MSC preparations were always cultured in parallel with each of the 31 HPL-supplements.

Osteoporosis and reduction of bone formation are major problems for elderly people. It is conceivable that MSC of osteoporotic bones have different characteristics. In contrast to such cell-intrinsic differences our study demonstrated that the osteogenic differentiation potential was reduced in each of the BM-MSC preparations if cultured with HPL of elderly donors. These results support previous observations by Abdallah and co-workers [28]. The analogy of decreased bone formation upon aging and reduced osteogenic induction with HPL of older donors indicates that soluble serum factors may contribute to decreased bone formation and osteoporosis. Further comparison of serum derived from osteoporotic and non-osteoporotic donors is necessary to demonstrate if systemically released compounds contribute to this disease.

So far, our analysis did not unravel relevant age-associated growth factors. Only PDGF-AB correlated moderately with osteogenic differentiation. It is conceivable that growth factors act synergistically and this might be addressed with mathematical models based on larger sample numbers. Other metabolites such as lipids and hormones may also be participating in functional regulation. It is also possible that HPL comprises microvesicles or exosomes which are involved in trafficking of miRNAs [57]. So far, it is unclear if the systemic feedback signals conversely influence cell intrinsic aging programs. We have previously demonstrated that MSC reveal specific gene expression changes [32] and DNA-methylation changes upon aging [36], [58], [59]. The precise mechanisms that govern cell-intrinsic age-associated epigenetic modifications are yet unclear, but theoretically they might be influenced by systemic factors. Further research will demonstrate if the effect of donor age of serum supplements is directly exerted by growth factors or other stimulatory molecules – or if it even feeds back on the cell-intrinsic aging program.

### Conclusion

Up to now serum supplements are still required for culture expansion of MSC. Our study supports the notion that age-associated soluble factors affect stem and progenitor cells – and this is also relevant for serum supplements in cell culture. Clinical trials with HPL-expanded MSC have already been performed and no adverse side effects were reported [60], [61]. However, clinical implications of our study should not be overstated. The clinical potency of MSC is not necessarily reflected by proliferation or *in vitro* differentiation potential – in the absence of reliable markers for clinical potency the optimal culture conditions have to be assessed for each application individually [13]. So far, the essential factors in serum supplements are not yet fully defined. Our study demonstrates that HPLs of younger donors enhance expansion rates of MSC, but the variance of proliferation is relatively high within HPL-age groups. Furthermore, MSC in clinical trials are often used at earlier passages (2^nd^ to 3^rd^ passage), whereas some of our results were generated with MSC at higher passages. HPL derived from younger donors may be advantageous for enhanced culture expansion, but the functional relevance can only be assessed in clinical trials. The results of this study do not have a clinical implication for the above mentioned reasons.

## Supporting Information

Figure S1 **Variability of human platelet lysates for MSC expansion.** MSC from bone marrow (A,C,E,G,I; n = 4) and adipose tissue (B,D,F,H,J; n = 3) were cultured in parallel with media supplemented with different HPLs. Proliferation was assessed after 7 days using the MTT assay. Signal intensity was normalized to the corresponding FCS control and analyzed in relation to platelet counts of HPL donors (A,B), or to the concentrations of PDGF-AB (C,D), TGF-β1 (E,F), bFGF (G,H) and IGF-1 (I,J).
(TIF)
