# Supplementary figures and images for: Donor Age of Human Platelet Lysate Affects Proliferation and Differentiation of Mesenchymal Stem Cells
Source: PLoS One. 2012 May 25;7(5):e37839. doi: 10.1371/journal.pone.0037839 (PMC3360602; doi:10.1371/journal.pone.0037839)

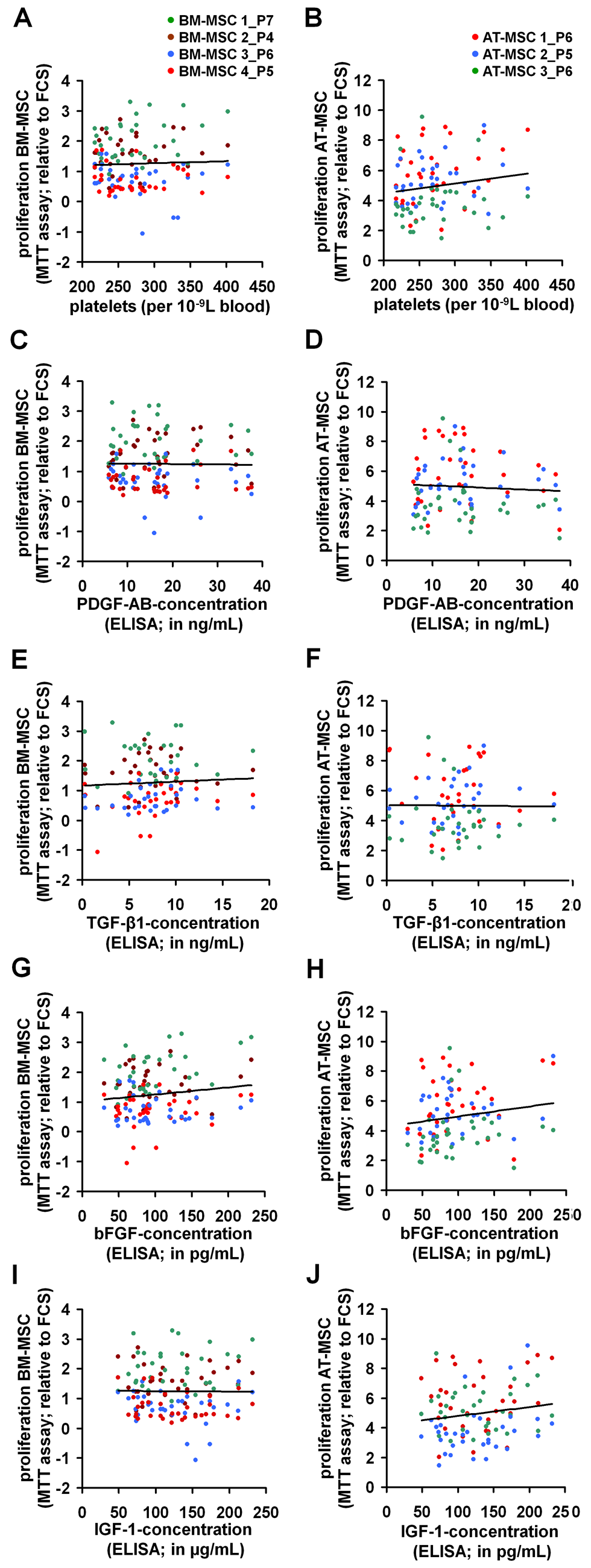

Supplement: Figure S1 — Variability of human platelet lysates for MSC expansion. MSC from bone marrow (A,C,E,G,I; n = 4) and adipose tissue (B,D,F,H,J; n = 3) were cultured in parallel with media supplemented with different HPLs. Proliferation was assessed after 7 days using the MTT assay. Signal intensity was normalized to the corresponding FCS control and analyzed in relation to platelet counts of HPL donors (A,B), or to the concentrations of PDGF-AB (C,D), TGF-β1 (E,F), bFGF (G,H) and IGF-1 (I,J). (TIF) [file pone.0037839.s001.tif]
